# Pilot Study on Next-Generation Sequencing Analysis of Vaginal Microbiota in Clinically Infertile Patients Treated with Probiotics

**DOI:** 10.3390/jcm13123420

**Published:** 2024-06-11

**Authors:** Li-Te Lin, Chia-Jung Li, Chia-Chun Wu, Li-Fei Pan, Kuan-Hao Tsui

**Affiliations:** 1 Department of Obstetrics and Gynaecology, Kaohsiung Veterans General Hospital, Kaohsiung 813, Taiwan; 2 Institute of Biopharmaceutical Sciences, National Sun Yat-sen University, Kaohsiung 804, Taiwan; 3 Department of Obstetrics and Gynaecology, National Yang-Ming University School of Medicine, Taipei 112, Taiwan; 4 Department of General Affair Office, Kaohsiung Veterans General Hospital, Kaohsiung 813, Taiwan; lfpan@vghks.gov.tw; 5 College of Finance and Banking, National Kaohsiung University of Science and Technology, Kaohsiung 824, Taiwan; 6 Department of Obstetrics and Gynecology, Taipei Veterans General Hospital, Taipei 112, Taiwan; 7 Department of Medicine, Tri-Service General Hospital, National Defense Medical Center, Taipei 114, Taiwan

**Keywords:** probiotics, ovarian aging, vaginal microbiota

## Abstract

**Background**: In this investigation, we aimed to understand the influence of oral probiotic supplementation on the vaginal microbiota of women preparing for assisted reproductive technology (ART) procedures. Given the importance of a healthy microbiome for reproductive success, this study sought to explore how probiotics might alter the bacterial composition in the vaginal environment. **Methods**: We recruited a cohort of 30 women, averaging 37 years of age (ranging from 31 to 43 years), who were scheduled to undergo ART. Using 16S ribosomal RNA (rRNA) sequencing, we meticulously analyzed the vaginal microbiota composition before and after the administration of oral probiotic supplements. **Results**: Our analysis identified 17 distinct microorganisms, including 8 species of Lactobacillus. Following probiotic supplementation, we observed subtle yet notable changes in the vaginal microbiota of some participants. Specifically, there was a decrease in Gardnerella abundance by approximately 20%, and increases in Lactobacillus and Bifidobacterium by 10% and 15%, respectively. Additionally, we noted a significant reduction in the Firmicutes/Bacteroidetes (F/B) ratio in the probiotic group, indicating potential shifts in the overall bacterial composition. **Conclusions**: These preliminary findings suggest that oral probiotic supplementation can induce significant changes in the vaginal microbiota of middle-aged women undergoing ART, potentially improving their overall bacterial profile. Future studies should consider a larger sample size and a narrower age range to validate these results. Investigating factors related to female hormone production could also provide deeper insights. Understanding the effects of probiotics on the vaginal microbiota in patients with ovarian aging may lead to personalized interventions and better reproductive outcomes.

## 1. Introduction

The female vagina serves as a crucial gateway to the reproductive system, where the vaginal microbiota plays a vital role in maintaining a dynamic balance and safeguarding the host against potential pathogens. Its influence on reproductive health extends to pregnancy outcomes, influencing the establishment and development of the newborn’s vital systems, including the skin, intestinal tract, urethra, and vaginal flora [1,2].

A healthy vaginal microbiota in women of childbearing age is predominantly dominated by *Lactobacillus* species (>70%), with *Lactobacillus crispatus*, *Lactobacillus iners*, *Lactobacillus gasseri*, and *Lactobacillus jensenii* being the top four species by abundance [3,4]. *Lactobacillus* exerts its positive effects on vaginal health by producing lactic acid and hydrogen peroxide, which help maintain an optimal vaginal pH. Factors such as estrogen levels, menstrual cycle, age, and pregnancy status influence the distribution and quantity of *Lactobacillus*, while external factors, including race, social and natural environments, living habits, and lifestyle choices, contribute to the diversity of the vaginal microbiota. Understanding the intricate interplay between the vaginal microbiota and its host can shed light on the development of tailored interventions to promote reproductive health and protect against potential health risks [4,5].

Compared to the normal vaginal microbiota in women of childbearing age, infertile women exhibit greater changes in the abundance of vaginal microbiota. Specifically, there is an increase in *Atopobium*, *Aerobicella*, and *Bifidobacterium*, while *Lactobacillus* and *Leuconostoc* show decreased abundance [4,6]. Bacterial vaginosis (BV), a common gynecological disease arising from the imbalance of microbial diversity in the reproductive tract, can lead to adverse pregnancy outcomes, including premature birth. While no definitive causal relationship between BV and infertility has been established, studies have indicated a higher incidence of BV in patients with tubal infertility compared to those with non-tubal infertility [7,8]. Kyono et al. conducted 16S rRNA sequencing on endometrial fluid and vaginal secretion samples from infertile patients and healthy women, revealing a considerable proportion of non-*lactobacillus*-dominated (NLD) microbiota in the endometrial fluid of infertile patients. Increasing the level of endometrial *Lactobacillus* to over 90% was proposed to potentially benefit the pregnancy outcomes of NLD infertile patients [9]. Campisciano et al. also performed 16S rRNA sequencing on the vaginal microbiota of patients with unexplained infertility, healthy women, and patients with known infertility and bacterial vaginosis. They found significant differences in microbial diversity among the samples, and the distribution of *Lactobacillus* varied between women with unexplained infertility and known infertility. These findings emphasize the importance of understanding the role of vaginal microbiota in fertility and reproductive health, paving the way for potential interventions to improve pregnancy outcomes in infertile women [10].

The main objective of this research was to evaluate how oral probiotics influence the structure and functional potential of the vaginal microbiota in infertile middle−aged patients. Utilizing 16S rRNA gene amplicon microbiota analysis, the researchers gained valuable insights into the potential effects of probiotics on the vaginal microbial ecosystem. Such understanding holds promise for enhancing reproductive health outcomes in this particular group of patients.

## 2. Materials and Methods

### 2.1. Study Design and Patients

The study was conducted at the Fertility Center, Kaohsiung Veterans General Hospital, Taiwan, spanning from January 2020 to December 2020. The study cohort comprised 30 infertile individuals of middle−age, all of whom were slated to undergo in vitro fertilization (IVF). The inclusion criteria were as follows: individuals aged between 30 and 45 years old, with a BMI ranging from 18 to 30 kg/m^2^, and no history of pelvic inflammatory disease or tubo-ovarian abscess. The exclusion criteria were as follows: individuals with current vaginitis, cervical lesions (such as erosion or polyps), cervical intraepithelial neoplasia (CIN), congenital uterine malformations, gynecologic cancer, recent supplementation with probiotics within the past 3 months, recent use of hormonal preparations within the past 3 months, polycystic ovary syndrome (PCOS), recurrent abortions, recent antibiotic use, history of contraceptive methods within the past 6 months, and recent history of infections.

Participants consumed probiotic capsules (Real-Free^®^ Plus Probiotics Capsule, Toppure Biotechnology Co., Ltd., Taipei, Taiwan) orally every day for at least 8 weeks prior to undergoing the IVF procedure. Each capsule contained 100 mg of Lactobacillus crispatus (LAT-1), 80 mg of Lactobacillus rhamnosus (RF-1), and 10 mg of Lactobacillus acidophilus (RF-2). Vaginal swabs were procured both before and subsequent to the course of oral probiotics capsule administration. Vaginal samples were collected by the same trained medical personnel in our reproductive center using standardized procedures. To reduce variability, samples were consistently collected during the mid-luteal phase of the menstrual cycle. All participants provided written informed consent, and their demographic information, menstrual characteristics, and clinical and laboratory data were retrieved from electronic medical records. Sample size calculations determined that a total of 30 participants in each study group would provide an optimal analysis with 80% power.

### 2.2. Ethics Statement

All procedures conducted in this study were approved by the Institutional Review Board of Kaohsiung Veterans General Hospital (KSVGH19-CT12-13) and were in accordance with the principles of the Declaration of Helsinki.

### 2.3. Sample Collection and DNA Extraction

The sample collection was conducted using a sterile sample collection kit (FLOQSwabs; COPAN, Murrieta, CA, USA). Vaginal swabs were collected at regular intervals and promptly placed in an icebox. Subsequently, the samples were transferred to a −80 °C freezer for DNA extraction and 16S rRNA gene sequencing. Genomic DNA extraction and library preparation for PacBio sequencing were performed by a company in Suzhou, China. DNA samples were quantified using a Qubit 3.0 Fluorometer (Invitrogen, Carlsbad, CA, USA). The amplification of full-length 16S rDNA was carried out using the forward primer 27F with the universal sequence “AGRGTTYGATYMTGGCTCAG” and the reverse primer 1492R with the universal sequence “RGYTACCTTGTTACGACTT.” Barcodes were introduced in a second round of amplification utilizing PacBio Barcoded Universal forward and reverse primers to generate libraries suitable for PacBio Sequel sequencing. The DNA input for generating amplicons using the full-length 16S Library Preparation kit ranged from 25 pg to 2.5 ng.

### 2.4. Illumina Sequencing

AllBio Science, Inc., Ltd. was tasked with conducting the gene sequencing and analysis for this study. The sequencing process is briefly as follows: DNA libraries were subjected to validation using the Agilent 2100 Bioanalyzer (Agilent Technologies, Palo Alto, CA, USA) and quantification using the Qubit 3.0 Fluorometer. Following this, the DNA libraries were multiplexed and loaded onto the PacBio Sequel instrument, following the guidelines provided by the manufacturer, Pacific Biosciences of California, Inc., Menlo Park, CA, USA.

### 2.5. Metagenomics Analysis

Circular consensus sequencing (CCS) reads underwent optimization, followed by the removal of low-quality sequences. Subsequently, the optimized reads underwent filtration via DADA2, involving denoising and chimera removal, to yield amplified sequence variants (ASVs). The 16S rRNA reference database used was Silva 138. Taxonomic analysis of ASVs’ representative sequences was conducted using the RDP Classifier (Ribosomal Database Program) Bayez algorithm. The community composition at various species classification levels was determined based on these analyses. Furthermore, alpha-diversity indices, including Shannon and Chao1, were calculated, along with community species abundance, and diversity-of-rarefaction curves and rank-abundance graphs were generated. These analyses reflect species richness and evenness based on the obtained ASV analysis results using the random sampling of sample sequences.

### 2.6. Gene Function Prediction

PICRUSt2 was used with the IMG database. The Tax4fun2 analysis utilized the official Ref99NR database, and R’s microeco package was employed for analysis, plotting, and metastat statistics. The FAPROTAX (V1.2.4) analysis was not limited to a specific database and was conducted using R’s microeco package for analysis, plotting, and metastat statistics.

### 2.7. Statistical Analysis of Species Differences

Statistical analyses included Wilcoxon’s signed-rank test and Welch’s *t*-test using R for inter-group species differences. MetagenomeSeq and ANCOM were used for taxonomic analysis, while the ALDEx2 and LEfSe packages were employed for species comparison. Spearman correlation and network analysis were conducted for the dominant species. Bubble plots were generated using ggplot2.

## 3. Results

### 3.1. Patients’ Characteristics and Pregnancy Outcomes

The participants had a mean age of 37.0 ± 3.8 years and a mean BMI of 23.3 ± 3.6 kg/m^2^. The average duration of infertility was 4.1 ± 3.0 years. Most participants had regular menstrual cycles, and the majority experienced primary infertility. The average duration of probiotic supplementation was 8.6 ± 1.5 weeks. Among the participants, 56.7% attained pregnancy subsequent to probiotic supplementation, with an average time to conception of 10.5 ± 12.4 weeks following the completion of the probiotic intervention (Table 1).

### 3.2. The Impact of Probiotics on the Diversity of the Female Reproductive Tract Microbiota

**Figure 1:**
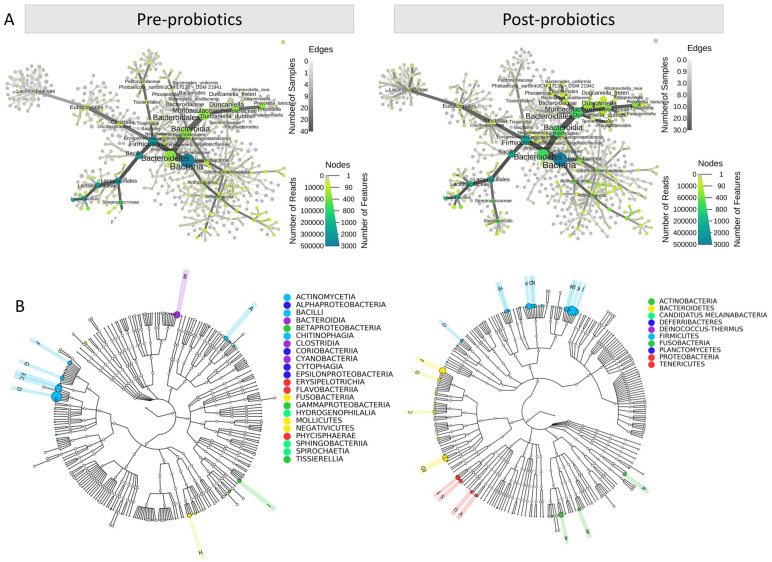
The heat tree graph illustrates the impact of probiotics on the diversity of the 16S microbiome. (**A**) When grouped, the circular heat tree represents the sequence abundance of different hierarchical taxa. The species-level heat tree displays hierarchies from the center to the outer layer, ranging from high (Kingdom) to low (Species). Sequence abundance is depicted through the node size, edge thickness, and color. (**B**) The graphical phylogenetic analysis offers visualized data of microbial and metagenomic genomes, presenting species evolutionary relationships, species abundance, and annotations, resulting in a more comprehensive and enriched depiction of the phylogenetic tree.

In this study, we investigated the impact of probiotics on the female reproductive tract using advanced next-generation sequencing techniques. By employing a heat tree analysis of 16S microorganisms, we gained rapid insights into the bacterial species distribution across different strata. The circular heat tree visually represented sequence abundance across different species classes, highlighting that the bacteria category had the highest number of sequences and the largest nodes and was marked by a distinctive brown color (Figure 1A). Additionally, Figure 1B illustrates the results of the graphical phylogenetic analysis, providing further evidence of the changes induced by probiotics over the 8-week period. Notably, the probiotic intervention led to a significant reduction in the complexity of the microbial community. This data visualization approach allowed for a more profound understanding of the changes in the microbial community and their potential implications for female reproductive health.

### 3.3. Richness, Diversity, and Differential Abundance of Reproductive Tract Microbiota

**Figure 2:**
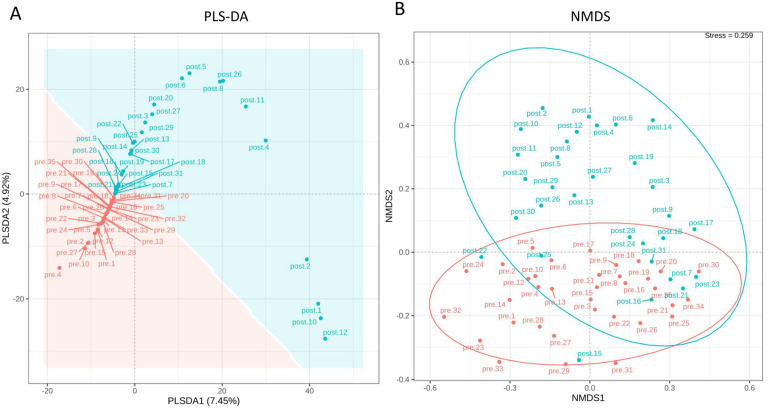
Partial Least Squares Discriminant Analysis (PLS−DA) and Non-metric Multidimensional Scaling (NMDS) were employed to analyze the impact of probiotics on the microbiota. (**A**) The PLS−DA plot reveals the distinct separation between pre-probiotics and post-probiotics microbiota. (**B**) NMDS analysis shows the spatial distribution of the genital microbiota in patients before and after probiotic administration. Each dot in the figure represents the microbiota profile of an individual patient in a reduced-dimensional space.

To better understand individual variations at the population level, PLS-DA and NMDS analyses were conducted. PLS-DA and NMDS analyses offer valuable tools to assess the composition and dynamics of the microbiota, contributing to a deeper understanding of the underlying mechanisms involved in probiotic-induced alterations in the reproductive tract. Regarding the genital microbiota samples, supervised PLS-DA revealed some separation of pre-treatment population clusters (depicted in red areas). However, the post-treatment samples showed a more significant dispersion along the PLS-DA2 axis (Figure 2A). Moreover, NMDS analysis indicated that pre-treatment samples tended to cluster along the NMDS1 axis, while post-treatment samples tended to cluster along NMDS2 (Figure 2B). These findings provide valuable insights into the impact of probiotics on the reproductive tract microbiota and suggest potential changes in the microbial community following probiotic administration. 

### 3.4. Assessment of Probiotics’ Influence on Vaginal Microbiota Composition

**Figure 3:**
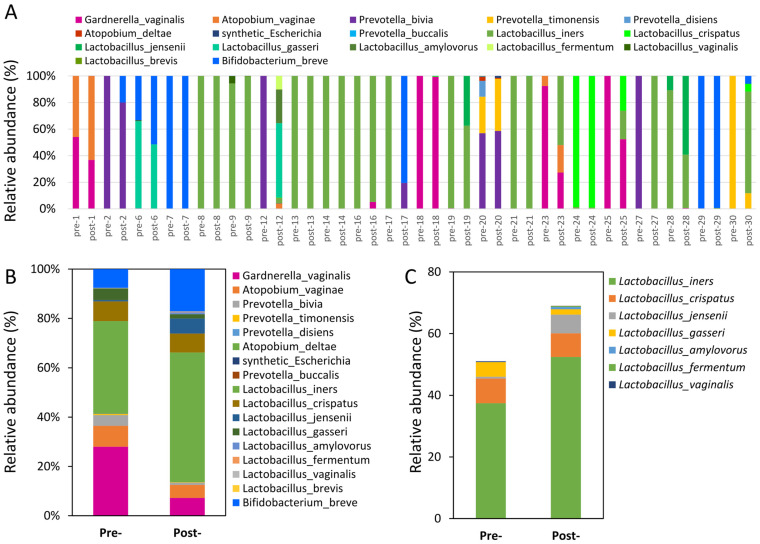
The vaginal microbiota composition pre- and post-intervention. (**A**) The relative abundance of the cervicovaginal microbiota of women before and after the test is shown for comparison across different patients. Additionally, (**B**) shows the genus-level abundance of *Lactobacilli*, while (**C**) shows the species-level abundance of *Lactobacillus* based on BLASTn analysis.

The impact of probiotics on the composition and diversity of vaginal microbiota was thoroughly investigated in this study (Figure 3A). Remarkable changes were observed in the abundance of specific bacterial species before and after the intervention. *Gardnerella* levels showed a significant decrease from 28% to 7.2%, while *Bifidobacterium* exhibited an increase from 7.6% to 17.2%. Furthermore, the beneficial bacteria *Lactobacillus* species were closely examined, revealing that *Lactobacillus iners* increased from 37.5% to 52.5%, whereas *Lactobacillus jensenii* experienced a substantial increase from 0.6% to 6.1% (Figure 3B). Our analysis indicated that the most substantial impact on the abundance of the *Lactobacillus* genus was observed before and after the intervention (Figure 3C). These findings suggest that the timing of the intervention is a critical factor in influencing the levels of *Lactobacillus* in the vaginal microbiota. Understanding the dynamics of *Lactobacillus* populations before and after the intervention can provide valuable insights into the potential mechanisms underlying the effects of probiotics on the vaginal microbiota. These findings highlight the significant alterations in the abundance of specific microbial species in response to the probiotic intervention, particularly with regard to *Lactobacillus* populations.

**Figure 4:**
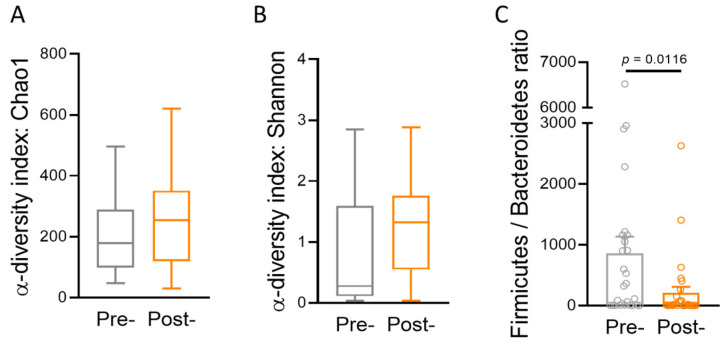
The analysis of the diversity of the cervicovaginal microbiota before and after the intervention. The alpha diversity of the cervicovaginal microbiota was assessed before and after the intervention. Alpha−diversity indices, including Chao1 (**A**), Shannon (**B**), and the Firmicutes/Bacteroidetes ratio (**C**), were used to measure species richness and evenness.

To evaluate the composition and distribution of different bacteria, we conducted a diversity analysis to assess bacterial richness, abundance, and evenness within each microbiota sample (Figure 4). The Chao1 and Shannon alpha−diversity indices did not show significant differences (Figure 4A,B), indicating that overall species richness and evenness remained relatively stable before and after the intervention. Notably, we observed a significant decrease in the Firmicutes/Bacteroidetes (F/B) ratio in the post-intervention group (Figure 4C). This shift in the microbiota composition is of interest, as the Firmicutes group contains genes related to glucuronidase regulation, which could impact estrogen metabolism. The high proportion of F/B flora has been associated with estrogen metabolic disorders. Therefore, it is plausible to speculate that probiotics may play a role in regulating obesity and estrogen metabolism in women.

### 3.5. Functional Pathways Predicted by PiCrust2 to Intervene in Probiotics

**Figure 5:**
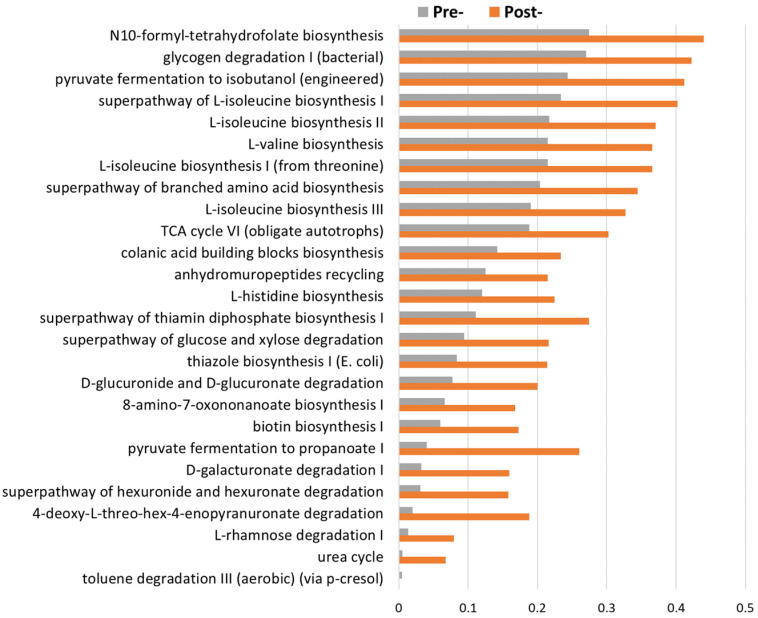
PICRUSt2 metabolic function prediction of bacterial flora. The presence and abundance of specific gene families in microbial communities were inferred by utilizing information from 16S rRNA gene sequences.

In addition to investigating the functional consequences of microbial diversity and structural variation, we sought to gain a deeper understanding of the potential effects of the taxonomic differences before and after the probiotic intervention. To achieve this, we performed metagenomic diversity predictions and carefully examined the differential abundance of KEGG genes and pathways. Notably, our findings revealed a remarkable increase in the metabolic activity of several microbial species following the probiotic intervention. This intriguing observation may be linked to the changes observed in the F/B ratio, as depicted in Figure 5. The comprehensive analysis of these functional implications further enhances the significance of our study in shedding light on the intricate interactions within the gut microbiota and their response to the probiotic intervention.

## 4. Discussion

This prospective study aimed to explore the impact of oral probiotics on the vaginal microbiota in infertile women. The study findings highlighted discernible alterations in microbial communities pre- and post-administration of oral probiotics. Notable changes included a reduction in the *Gardnerella* population, an elevation in *Lactobacillus* species, and an augmentation in metabolic activity subsequent to the oral probiotic intervention. The capacity of probiotics to potentially influence reproductive processes positively could be attributed to their ability to modulate microbiota compositions, regulate metabolism, strengthen the epithelial barrier, and boost immune functionality. Previous studies have suggested their role in promoting reproductive health [11]. Our research confirms the ability of oral probiotics to regulate microbiota composition and improve metabolic activity, thus supporting their beneficial effects on reproductive health.

The microbiota within the female genital tract has been extensively investigated for its potential associations with fertility prospects and ART outcomes [12,13,14,15]. Notably, the presence and abundance of specific *Lactobacillus* species, particularly *Lactobacillus crispatus*, have been implicated in enhancing reproductive success. Conversely, pathogens such as *Gardnerella vaginalis* have been linked to unfavorable reproductive outcomes [16,17]. A prospective cohort study conducted by Karaer et al. enrolled 223 women undergoing vaginal sample collection prior to IVF treatment. This study revealed that individuals who did not achieve pregnancy displayed a lower relative abundance of *Lactobacillus* (67.71% vs. 79.72%), alongside elevated relative levels of *Streptococcus* (7.81% vs. 2.28%) and *Gardnerella* (9.40% vs. 5.56%), as compared to their pregnant counterparts [13]. In a similar vein, another prospective cohort study involving 130 infertile patients undergoing IVF treatment showcased a significantly diminished clinical pregnancy rate in those with abnormal vaginal microbiota compared to those with a normal microbial profile (9% vs. 44%) [14]. Furthermore, the genital microbiota has emerged as a potential predictor of ART outcomes [18,19,20,21]. In a prospective cohort study by Koedooder et al., a cohort of 303 women were subject to pre-IVF vaginal sampling, revealing that individuals harboring an unfavorable vaginal microbiota composition exhibited a sevenfold lower likelihood of achieving pregnancy compared to those with a favorable profile [18]. Consistent findings indicated that infertile women, particularly those encountering recurrent implantation failure (RIF), exhibited decreased *Lactobacillus* growth and heightened *Gardnerella* growth, distinguishing them from their fertile controls [20,22,23]. *Gardnerella* species are linked to chronic inflammation and altered immune responses in the vaginal environment. Their presence can induce the production of pro-inflammatory cytokines, resulting in local inflammation that may impact the reproductive tract and impair fertility. Additionally, *Gardnerella* can form biofilms on the vaginal epithelium, shielding the bacteria from host immune defenses and antibiotic treatments. These biofilms contribute to persistent infections and chronic vaginal diseases, which can further compromise fertility. Together, the findings from these studies and our current investigation may suggest the potential of oral probiotics to enhance reproductive outcomes by fostering *Lactobacillus* proliferation and curbing *Gardnerella* abundance. Such probiotic interventions might potentially yield favorable results among infertile individuals, particularly those with RIF. However, despite the promising implications of probiotics’ influence on vaginal health derived from studies [24,25,26], their direct impact on enhancing reproductive success remains inconclusive [27,28,29]. A randomized controlled trial involving 340 infertile women undergoing frozen embryo transfer and divided into a study group (receiving daily intravaginal lactobacilli tablets for six days) and a control group (no probiotic supplementation) demonstrated comparable biochemical and clinical pregnancy rates between the groups, with a notable reduction in the miscarriage rate within the study group [27]. However, questions surrounding the short duration of probiotic supplementation cast doubt on the regimen’s efficacy. Large-scale randomized controlled trials are imperative to definitively establish the relationship between oral probiotics, the genital microbiota, and reproductive outcomes.

In addition to assessing microbial abundance, the impact of microbiome richness and diversity on reproductive outcomes has garnered attention, although definitive conclusions have yet to be reached [16,17]. Some studies have suggested a negative association between heightened microbiome richness or diversity and reproductive outcomes [21,30,31,32], while others have reported negligible effects on reproductive outcomes [22,33]. Freitas et al. conducted an extensive analysis comparing the vaginal microbiomes of healthy pregnant women (*n* = 182) at 11–16 weeks of gestational age with those of non-pregnant women (*n* = 310). Their findings illuminated that the richness and diversity of vaginal microbiomes were notably reduced in healthy pregnant women in comparison to their non-pregnant counterparts [31]. However, in a prospective case–control study encompassing 31 patients undergoing ART with a frozen-thawed single euploid blastocyst, the potential influence of the vaginal microbiota on IVF outcomes was explored. The study unveiled no statistically significant disparities in terms of alpha diversity or beta diversity between women who achieved pregnancy and those who did not [33]. In the present study, we assessed the Shannon diversity index and the Chao1 diversity index before and after oral probiotic administration. Notably, our findings indicated no significant alterations in these diversity indices (Figure 4). However, a significant decrease in the F/B ratio was observed following oral probiotic administration (Figure 4). This parameter, recognized for its pivotal role in maintaining intestinal homeostasis, has been linked to various conditions, such as obesity, inflammatory bowel disease, and irritable bowel syndrome [34,35]. Yet, despite the growing knowledge regarding the F/B ratio’s impact on the gut microbiota, its significance within the vaginal microbiota remains largely unexplored and necessitates further investigation.

Moreover, the present investigation has illuminated the capacity of oral probiotics to augment the metabolic dynamics of the vaginal microbiota (Figure 5). Fu et al. conducted a study to compare individuals with unexplained RIF with those who got pregnant within the initial frozen embryo transfer cycle. They looked at differences in both the vaginal bacteria and metabolic profiles between these two groups. Significant differences were present in both the vaginal microbiota and metabolomic compositions between the two cohorts. Specifically, diminished *Lactobacillus* abundance, as well as reduced levels of glycerophospholipids and benzopyran, were observed in the RIF cohort [30]. Importantly, it is plausible that the observed shifts in metabolite profiles emanate from or contribute to the distinct microbiota compositions. This underscores the intricate interplay between the vaginal microbiota and metabolites, whereby alterations in one domain might cause changes in the other. In line with this concept, Oliver et al. demonstrated associations between the cervicovaginal microbiota composition and the metabolic profile in healthy pregnancies [36]. 

Building upon these premises, our study has identified variations in the microbiota composition following oral probiotic administration, leading to consequent changes in metabolic activities. This supports the hypothesis that oral probiotics, through the modulation of microbiota configurations and associated metabolic pathways, could potentially offer advantageous implications for reproductive well-being. The vaginal microbiota plays a crucial role in maintaining reproductive health through the production of various metabolites, such as lactic acid, short-chain fatty acids (SCFAs), and bacteriocins. These metabolites lower vaginal pH, inhibit the growth of pathogenic bacteria, and maintain a balanced microbial ecosystem, which is essential for optimal reproductive function. Additionally, specific vaginal bacteria are involved in the metabolism of estrogens and other hormones. By modulating hormonal levels, these bacteria significantly influence the vaginal environment and reproductive processes. The interaction between microbial metabolites and the host immune system further underscores the importance of the vaginal microbiota. These metabolites modulate local immune responses, affecting inflammation and susceptibility to infections—factors critical for reproductive success. Furthermore, the vaginal microbiota may enhance the bioavailability and absorption of essential nutrients, impacting overall metabolic health. This enhancement ensures that the body has the necessary resources for successful conception and pregnancy. Collectively, these functions highlight the vital role of the vaginal microbiota in supporting reproductive health through complex metabolic and immunological interactions. Nonetheless, it is imperative to conduct thorough and large-scale studies to confirm the potential benefits of oral probiotics for reproductive health. 

The current study had several limitations. Firstly, the small number of participants limited the statistical reliability and validity of the findings. This also restricted the generalizability of the study’s conclusions. Secondly, the lack of a true control group, consisting of participants not receiving probiotic supplementation, made it challenging to definitively establish the association between probiotic intake and vaginal microbiota composition. Thirdly, the study only observed short-term effects during the treatment period, lacking long-term follow-up to assess the sustained impact of probiotics on the vaginal microbiota composition. Additionally, potential confounding variables such as dietary habits, lifestyle factors, and concurrent medication use were not adequately addressed or controlled for in the study design, potentially influencing the observed outcomes. Furthermore, the study primarily focused on microbial abundance and metabolic activity, neglecting other relevant outcome measures such as changes in inflammatory markers, vaginal pH, and clinical endpoints like pregnancy rates, which could offer a more comprehensive assessment of probiotics’ effects. One of the study’s limitations is the need to include data on the vaginal microbiota of reproductive−age women who did not consume probiotics, both before and after the intervention, to provide a baseline for comparison. Lastly, the study predominantly included infertile middle−aged women, limiting the generalizability of the findings to broader populations of women with varying demographic characteristics and reproductive health statuses. 

In future studies, additional conditions should be incorporated, including control participants who do not receive probiotic supplements, to better establish the association between probiotic intake and changes in vaginal microbiota composition. It is essential to control for potential confounding variables such as dietary habits, lifestyle factors, and concurrent medications. Furthermore, including clinical endpoints such as markers of inflammation, changes in vaginal pH, and pregnancy rates would provide a more comprehensive assessment of the effectiveness of probiotics. We acknowledge these limitations and suggest incorporating these considerations in future research to enhance the robustness of the findings. Including control groups and addressing potential confounders will help to more accurately attribute observed changes to probiotic supplementation. Additionally, evaluating clinical endpoints will offer a more holistic understanding of the impact of probiotics on reproductive health. 

## 5. Conclusions

Our study focused on infertile participants and revealed that the level of *Gardnerella* in the vaginal microbiota of infertile women is relatively high. However, taking probiotics can lead to a clear reduction in *Gardnerella* levels and an increase in *Bifidobacterium* and *Lactobacillus* levels. While these findings are promising, larger sample sizes are needed to further validate these changes. Nevertheless, our study highlights the potential of probiotics to modify gut microbiota, which, in turn, may indirectly impact the composition of the reproductive tract flora. Understanding these factors is crucial for promoting research on microbiome modulation and the development of treatments for vaginal disorders. It is important to consider these factors when interpreting conflicting evidence on the use of probiotics for promoting vaginal health.

## Figures and Tables

**Table 1:** Patients’ characteristics and pregnancy outcomes.

Items	Data
Age (years)	37.0 ± 3.8
Body weight (kg)	61.9 ± 12.5
BMI (kg/m^2^)	23.3 ± 3.6
Infertility duration (years)	4.1 ± 3.0
Menstruation cycle	
Regular	83.3%
Irregular	16.7%
Menstruation period (days)	29.0 ± 2.3
Menstruation length (days)	6.0 ± 1.6
Type of infertility	
Primary	63.3%
Secondary	36.7%
Duration of probiotic supplementation (weeks)	8.6 ± 1.5
Pregnancy rate (%)	56.7%
Time to pregnancy following probiotic intervention (weeks)	10.5 ± 12.4

## Data Availability

No new data were created or analyzed in this study. Data sharing is not applicable to this article.
